# Maize Bran Particle Size Governs the Community Composition and Metabolic Output of Human Gut Microbiota in *in vitro* Fermentations

**DOI:** 10.3389/fmicb.2020.01009

**Published:** 2020-05-25

**Authors:** Riya D. Thakkar, Yunus E. Tuncil, Bruce R. Hamaker, Stephen R. Lindemann

**Affiliations:** ^1^Whistler Center for Carbohydrate Research, Department of Food Science, Purdue University, West Lafayette, IN, United States; ^2^Food Engineering Department, Ordu University, Ordu, Turkey

**Keywords:** maize bran, particle size, milling, upper gastrointestinal digestion, short-chain fatty acids, butyrate, 16S rRNA sequencing

## Abstract

Differences in the chemical and physical properties of dietary fibers are increasingly known to exert effects on their fermentation by gut microbiota. Here, we demonstrate that maize bran particle size fractions show metabolic output and microbial community differences similar to those we previously observed for wheat brans. As for wheat brans, maize bran particles varied in starch and protein content and in sugar composition with respect to size. We fermented maize bran particles varying in size *in vitro* with human fecal microbiota as inocula, measuring their metabolic fate [i.e., short-chain fatty acids (SCFAs)] and resulting community structure (via 16S rRNA gene amplicon sequencing). Metabolically, acetate, propionate and butyrate productions were size-dependent. 16S rRNA sequencing revealed that the size-dependent SCFA production was linked to divergent microbial community structures, which exerted effects at fine taxonomic resolution (the genus and species level). These results further suggest that the physical properties of bran particles, such as size, are important variables governing microbial community compositional and metabolic responses.

## Introduction

The human gut houses trillions of microorganisms, and their physiological importance in human health is becoming increasingly understood. The microbial associates that live in and on the human body are collectively referred to as its microbiota (Clemente et al., 2012). The human gut is thought to be populated with ∼38 trillion microbial cells (Sender et al., 2016), which code for approximately 100-fold more unique genes as the human genome (Ley et al., 2006), composing microbial communities important enough to be referred to as a ‘forgotten organ’ (O’Hara and Shanahan, 2006). Microbiome composition and function are known to be strongly driven by environmental factors such as diet, host health status (including differences between healthy states, such as pregnancy), xenobiotic exposure, and antibiotic intake (Dethlefsen et al., 2008; Björkholm et al., 2009; Turnbaugh et al., 2009; Koren et al., 2012). Of the above factors, diet is known to strongly govern the population sizes of different gut-dwelling microbial species (Ley et al., 2008).

Colonic microbes ferment carbohydrates and proteins that escape human digestion in the small intestine (Koropatkin et al., 2012), metabolizing these resistant dietary glycans (i.e., dietary fibers) into short-chain fatty acids [SCFAs, principally acetate, propionate, and butyrate (Cummings et al., 1987)] that are largely absorbed by the host (Ohira et al., 2017). Dietary fiber intake is strongly linked with maintenance of diverse microbiota; diversity is drastically reduced in mice fed a low-MAC (microbiota-accessible carbohydrates) diet over a few generations compared with high-MAC-fed controls (Sonnenburg et al., 2016). Variation in total dietary fiber intake is also linked to changes in the abundances of microbial species due to their different fiber-metabolizing capacities (Sonnenburg et al., 2010). However, it is increasingly clear that microbiome effects depend not only on total dietary fiber consumption but also upon the diverse structures of fibers consumed. Specificity in consumption of dietary fiber structures by microbial species is known to govern their population sizes in the gut (Dominianni et al., 2015). Differences among organisms in their ability to consume different dietary fibers suggests that fine fiber structure is a potential mechanism for predictably modulating the gut microbiota.

Widespread consumption of the high-sugar/high-fat and low-fiber Western diet is linked to changes in the composition and metabolic activity of these human gut microbiomes compared with those fed more-traditional diets (Turnbaugh et al., 2009). In Western countries, approximately 50% of dietary fiber intake is cereal-derived (mostly from wheat, maize, oat, barley, and rye) (Lambo et al., 2005; Gebruers et al., 2008). However, one often-overlooked correlate of the overall reduction in dietary fiber intake as diets westernize is a reduction in particle sizes of grains consumed. As cultures shift from more traditional milling methods to mechanized steel roller mills and automated sifting devices, the efficiency of producing fine particles increases (Teague, 1952). Additionally, separation of different product streams (i.e., germ, bran, and endosperm fractions) is now possible using modern milling methods, whereas flours previously included the entire content of cereal grain unless the flour was sieved post-milling (Teague, 1952). Modern refined flour is comprised mainly of fine endosperm particles, and germ and bran are removed during the milling process. In some countries, such as the United States, production of “whole” flour typically involves brans being milled separately to small particle sizes and added back to refined flour. This reduction in the grain particle with westernization may, in part, explain microbiome differences among Western and non-Western populations (Cordain et al., 2005).

The effects of particle size on microbial fermentation of dietary fibers are not well-understood. Previous studies have focused on comparing coarse and fine wheat brans to evaluate size effects on transit time and laxation (Kirwan et al., 1974; Brodribb and Groves, 1978; Jenkins et al., 1999). Brodribb and coworkers measured stool weight after consumption of coarse and fine wheat brans and found larger increases with coarse bran than fine (Brodribb and Groves, 1978). They presented three possible reasons for this effect: first, that coarser brans have higher water-holding capacity; second, that coarser brans may be less digestible by gut bacteria; and third, that larger fiber particles might trap more gas produced by gut bacteria, thus increasing stool bulk (Walker, 1947). With respect to microbial fermentation, Stewart and Slavin found that coarse (average size = 1239 μm) and fine (average size = 551 μm) wheat brans elicited different SCFA production (Stewart and Slavin, 2009) in *in vitro* fermentation by fecal microbiota. In mice, wheat bran fractions differing in particle size have been shown to divergently influence fecal microbiome structure, fat excretion, and immune and inflammatory responses (Suriano et al., 2017).

Recently, we reported that different size fractions of wheat bran particles selected for different microbial communities and fermented to different metabolic outcomes (Tuncil et al., 2018b). The selective effects of bran particles were observed at very fine taxonomic levels, even within operational taxonomic units (OTUs, computational analogs of species) identified from the same genera. These effects were likely responsive to differences in chemical composition among differently-sized particles. To determine whether size effects were constrained to wheat bran particles or whether size-dependence was a more general property of bran fermentation by gut microbiota, we milled maize brans into similar size ranges and performed the same fermentation experiment. Here, we demonstrate that particle size effects occur in *in vitro* fermentation of maize brans by fecal microbiota.

## Materials and Methods

### Maize Brans

Maize bran particles were a gift from Bunge Milling (St. Louis, MO, United States). To obtain size fractions of maize bran, particles were sieved using a sieving machine (Portable Sieve Shaker Model RX-24, both sieving machine and screens were from W. S. Tyler Combustion Engineering, Inc., Mentor, OH, United States). The biggest size fraction obtained on sieving was 500 to 850 microns. This maize bran was further milled using a cyclone mill (FOSS North America, Eden Prairie, MN, United States) to obtain smaller particle sizes, to ensure that all the size fractions were derived from the same parent source of maize bran. We obtained four sizes of maize bran particles: (1) 180–250 μm (2) 250–300 μm (3) 300–500 μm and (4) 500–850 μm using sieving machines and screens both from W. S. Tyler Combustion Engineering, Inc., Mentor, OH, United States. These size fractions were used for further experiments.

### Upper Gastrointestinal Digestion of Brans

We simulated passage through the upper gastrointestinal (GI) tract of the different sizes of maize bran particles obtained after milling and sieving through *in vitro* digestion as previously described (as Method A) (Yang et al., 2013). We chose this method for simulated digestion because we recently determined that this method is generally more effective at removal of starch content for maize (Tuncil et al., 2018a). During the upper GI digestion, the maize bran particles were treated with pepsin, pancreatin and amyloglucosidase for 30 min, 6 and 6 h respectively. This was followed by dialysis for 36 h and freeze drying prior to *in vitro* fermentation.

### Neutral Monosaccharide Composition Analysis

We analyzed neutral monosaccharides from different maize bran particle sizes post-upper GI tract digestion using gas chromatography on a capillary column (SP2330, Supelco, Bellefonte, PA, United States) coupled with mass spectroscopy (GC–MS; models 7890A gas chromatograph and 5975C inert mass selective detector, Agilent Technologies, Inc., Santa Clara, CA, United States) as described previously (Tuncil et al., 2018a). Helium was used as carrier gas.

### Total Starch and Protein Contents Analyses

After upper-GI tract digestion, total starch, and protein contents of the maize bran having different particle sizes were analyzed. The total starch contents were quantified spectrophotometrically using the total starch assay kit (K-TSTA; Megazyme International, Wicklow, Ireland), according to the manufacturer’s instructions. Protein contents of the samples were determined by the Dumas method (N × 6.25) using a LECO TruMac nitrogen analyzer (LECO Corporation, St. Joseph, MI, United States) (Tuncil et al., 2018a).

### *In vitro* Fermentation

We performed *in vitro* anaerobic fermentations of maize bran particles of distinct sizes, using fecal microbiota as inoculum, within an anaerobic chamber (Coy Laboratory Products, Inc., Grass Lake Charter Township, MI, United States). Different sizes of maize bran samples (1) 180–250 μm (2) 250–300 μm (3) 300–500 μm and (4) 500–850 μm were weighed (44 ± 1 mg) and placed in Balch tubes (Chemglass Life Sciences, Vineland, NJ, United States). The anaerobic chamber was supplied with the following gas mix: 90% N_2_, 5% CO_2_ and 5% H_2_. Tubes containing media and either maize bran particles, FOS (a positive fermentation control), or no carbon source (blank) were placed in the anaerobic chamber overnight to remove oxygen; resazurin was used as the oxygen indicator. For all experiments, as previously we used phosphate-buffered gut mineral medium containing trace elements (8.0 mM NaCl, 6.3 mM KCl, 3.3 mM urea, 3.3 mM NH_4_Cl, 0.7 mM Na_2_SO_4_, 40 mM sodium phosphate buffer (pH 7.0), 1 mg resazurin, 0.25 g/L cysteine HCl, 333 μM CaCl_2_, 492 μM MgCl_2_, and 1X P1 metals and trace elements) (Tuncil et al., 2018a).

On the day of inoculation, we added 4 ml of gut mineral medium to all the tubes with different sizes of maize bran (44 mg per tube), the blank tubes and a fermentation positive control containing fructooligosachharides (FOS; Sigma-Aldrich, St. Louis, MO, United States). We collected and pooled fecal samples from three healthy donors (two males, 27 and 32 years old, respectively; one female, 31 years old, all three were omnivores) and pooled as previously described (Tuncil et al., 2018b). We pooled the fecal samples as pooling provides a diverse pool of species that is not limited by an individual’s idiosyncratic gut microbiome (Tuncil et al., 2018b). A previous study has shown that pooling of fecal microbiota shows similar microbial responses compared to similar experiments using individual donors (Aguirre et al., 2014). Human stool collection and use protocols were reviewed and approved by Purdue University’s Institutional Review Board (IRB Protocol #1701018645). The donors had been following their habitual diets and had not taken any antibiotics at least 12 weeks before the study began. To prevent the loss of bacterial viability, the fecal samples were collected and sealed as quickly as possible in 50 mL Falcon tubes, kept on ice, and rapidly transferred to the anaerobic chamber. We inoculated the bran particles and media with fecal slurry within 2 h of receiving the fecal microbiota from donors. We mixed the fecal samples and gut mineral media in the ratio 1:10 (w/v), and then filtered through four layers of cheese cloth. After filtration, the we pooled the fecal slurries from individual donors in equal ratios (Aguirre et al., 2014). We added 0.4 ml of the pooled fecal slurry mix to each Balch tube, closed the tubes with butyl rubber stoppers, sealed them with aluminum seals (both from Chemglass Life Sciences, Vineland, NJ, United States), and incubated them at 37°C in a shaking incubator (Innova 42, New Brunswick Scientific, Edison, NJ, United States) at 150 rpm at an approximately 45° angle. Fermentations were performed in triplicate.

### Total Gas, pH, and SCFA Measurements

At 6, 12, 24, 36, and 48 h time points after inoculation, we measured total gas production from fermentation as overpressure using a graduated syringe and passing a needle through the rubber stopper prior to unsealing the tubes. For pH measurements, the supernatant was transferred to a separate 15 ml Falcon tube and measured using a pH meter. We collected two aliquots from each tube, one for SCFA (0.4 ml) and other for DNA extraction (1 ml) and stored the aliquots at −80°C. An internal standard (157.5 μl of 4-methyl valeric acid, 1.47 ml of 85% phosphoric acid, 39 mg of copper sulfate pentahydrate in a total volume of 25 ml) was immediately added to the SCFA aliquots samples before vortexing.

We measured SCFAs as previously described (Tuncil et al., 2018b). Briefly, 4 μl of the supernatants were analyzed on a fused-silica capillary column (Nukon^TM^, SUPELCO No: 40369-03A, Bellefonte, PA, United States) using a gas chromatograph (GC-FID 7890A, Agilent Technologies, Inc.) (Tuncil et al., 2017). We used 4- methylvaleric acid (Fisher Scientific) as an internal standard and acetate, propionate, and butyrate (Fisher Scientific, Hampton, NH, United States) to generate standard curves.

### DNA Extraction

The FastDNA SPIN^®^ kit for Feces (MP Biomedical, Santa Ana, CA, United States; product code: 116570200) was used for DNA extraction. We followed the protocol as described in the user’s manual with the following modifications: as a first step before following the protocol, we thawed the sample, centrifuged the samples at 13,000 rpm for 10 min, and discarded the supernatant. We then added 825 μl of phosphate buffer as in the first step of protocol to resuspend the pellet by pipetting it multiple times. We followed the user’s manual for the rest of the protocol.

### 16S rRNA Sequencing

We amplified the V4–V5 region of 16S rRNA gene by PCR using the universal bacterial primers: 515-FB (GTGYCAGCMGCCGCGGTAA) and 926-R (CCGYCAATTYMTTTRAGTTT) as previously described (Tuncil et al., 2018b). The PCR cycle parameters were: (1) initial denaturation: 95°C for 5 min, (2) denaturation: 98°C for 20 s, annealing: 60°C for 15 s, extension: 72°C for 30 s for each of 22 cycles, and (3) final extension: 72°C for 10 min. After this, the samples were held at 4°C. From this amplified product we removed the leftover unattached primers, primer-dimers and dNTPs using the AxyPrep Mag PCR Cleanup Kit (Corning, Inc., Corning, NY, United States), which was followed by barcoding the PCR product using the TruSeq dual indexing approach for five cycles and a final clean up step to remove excess primers and primer dimers, if any, all as previously described in detail (Tuncil et al., 2018b). We quantified the cleaned, barcoded amplicons using Qubit dsDNA HS Assay Kit (Invitrogen, Carlsbad, CA, United States), and grouped and pooled amplicons according to similarities in concentration. The Purdue Genomics Core Facility performed quality control for pools by running 1 μl of each pool on an Agilent Bioanalyzer with a high sensitivity chip (Agilent, Santa Clara, CA, United States) and then quantified the pool loading via the KAPA Library Quantitation Kit for Ilumina platforms. Sequencing was then performed using an Ilumina MiSeq run with 2 × 250 cycles and V2 chemistry (Ilumina, Inc., San Diego, CA, United States).

### Sequence Processing and Community Analysis

Sequences were processed as previously described (Tuncil et al., 2018b). Briefly, we used mothur v. 1.39.5 according to the MiSeq SOP^1^ (accessed 12/1/2017) with the following modifications: sequences were screened with a maximum length of 411 and maximum homopolymer length of 9. For classification, we used the mothur-formatted version of the RDP training set v. 16 to which species epithets had been added and classified sequences at a bootstrap cutoff of 95%. We removed sequences classified as chloroplasts, mitochondria, or within *Eukarya*. Groups were subsampled to 2462 reads to normalize sampling effort across samples. Ecological α-diversity metrics were calculated using the nseqs, coverage, sobs, chao, simpsoneven, invsimpson, and shannon calculators in mothur. β-Diversity metrics were generated using the braycurtis, thetayc, and jclass calculators and plotted using the pcoa calculator in mothur. Linear discriminant analysis was performed using LEfSe v. 1.6 at an LDA cutoff of 3.5.

### Statistical Data Analysis

We used GraphPad Prism version 8.0.1 (GraphPad Software, Inc., La Jolla, CA, United States) for statistical analysis. ANOVA (Analysis of Variance) at α = 0.05 was performed to identify if there were any significant differences amongst treatments. The ANOVA test was followed by Tukey’s multiple comparison test at α = 0.05 for SCFA analysis and neutral monosaccharide, starch, and protein composition analyses to identify if means differed significantly among treatments. The PCoA plot was generated using R, version 3.5.1, using the vegan package.

## Results

### SCFA Production From Maize Bran Fermentation Is Particle Size-Dependent

**FIGURE 1:**
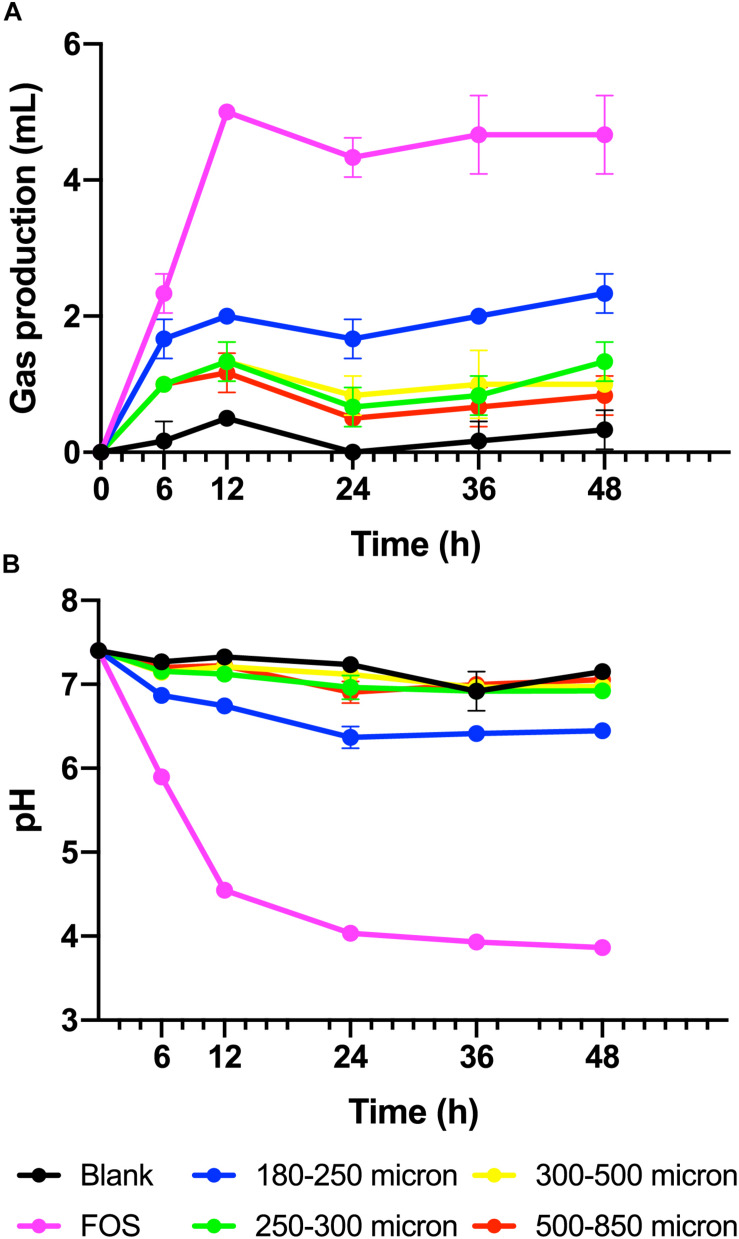
**(A)** Gas produced from maize brans varying in size by fecal microbiota in *in vitro* fermentation over time, measured as overpressure in Balch tubes after fermentation for 6, 12, 24, 36, and 48 h. **(B)** Culture pH at time points in fermentation of maize brans varying in size by fecal microbiota. The negative control (blank) contained no carbon source, the positive control (FOS) contained an equivalent amount of fructooligosaccharide. Error bars represent standard error of the mean.

**FIGURE 2:**
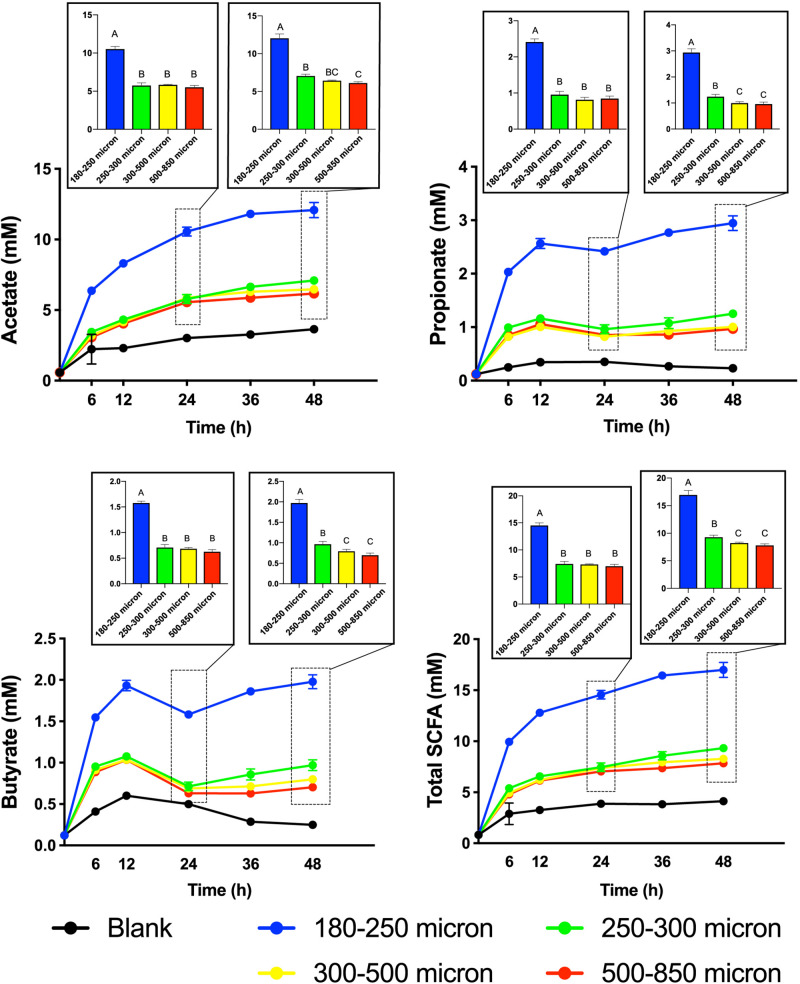
Short-chain fatty acid (SCFA) production from maize bran particles varying in size over the fermentation time course. The x-axis represents time in hours and y-axis represents concentration of SCFA in mM. Blanks contain no carbon source. Bar graphs represent the concentrations of SCFAs at 24 and 48 h. Total SCFA represents the sum of the acetate, propionate, and butyrate concentrations (mM). Error bars represent the standard error of the mean of triplicate cultures. Values of bars with same letters are not significantly different using Tukey’s test at *p* < 0.05; different letters represent statistically significant differences.

To test our hypothesis that the fermentation of maize bran is particle size-dependent, we performed *in vitro* fermentation with maize brans milled to a range of particle sizes. After *in vitro* fermentation, we measured the total gas production, pH, and SCFA composition at 0, 6, 12, 24, 36, and 48 h. Fast-fermenting FOS was used as a positive control. Figure 1 shows changes in pH and gas production over the fermentation time course; except for the 180–250 μm cultures, which were significantly elevated in both gas and acid production, cultures consuming different sizes of particles were insignificantly different from one another. Except for the FOS positive control, all cultures remained above a pH of 6.0 throughout the time course. Figure 2 shows significant differences among different size fractions in acetate, propionate, butyrate, and total SCFAs produced over time. Acetate, propionate, and butyrate were produced in a roughly 75:13:12 ratio, respectively, across all size fractions and time points. The smallest size fraction (180–250 μm) was much more extensively fermented, eliciting the highest amount of all three SCFAs (acetate, propionate, and butyrate) at all time points. The other three sizes (250–300, 300–500, 500–850 μm) produced less than half the amount for all the SCFAs compared to the smallest fraction. Even given the much smaller differences in SCFA production among the three larger sizes, acetate, propionate, and butyrate were all significantly higher in the 250–300 μm size cultures. Fermentation of the 300–500 and 500–850 μm fractions produced very small amounts of metabolic products (i.e., gas, SCFAs), which were insignificantly different from one another and only approximately double the concentrations observed in no-carbon controls. These data strongly suggested that the fate of particles was different among size fractions, most notably due to greater rates and extents of fermentation by microbiota for the smallest fraction.

### Different Particle Sizes of Maize Bran Selected for Distinct Microbial Community Structures

**FIGURE 3:**
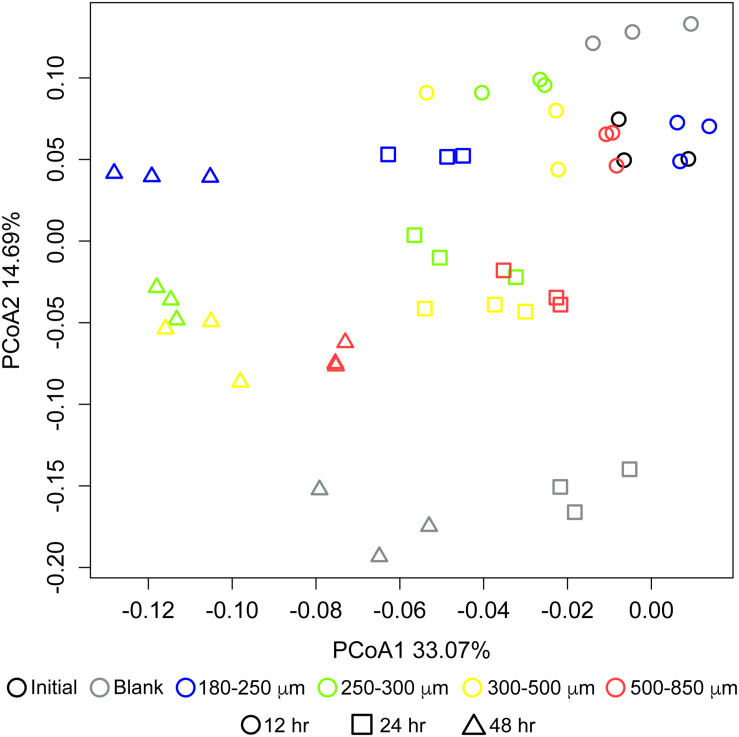
Differences in microbial community structure between maize bran-fermenting fecal microbiota from three healthy donors. Principal component analysis (PCoA) of communities fermenting different maize bran size fractions were determined by 16S rRNA amplicon sequencing, and β-diversity metrics were based upon Bray–Curtis dissimilarity calculated using from OTU relative abundances after 12, 24, and 48 h of *in vitro* fermentation. Initial represents the fecal community in the inoculum. Blank represents a no-carbon control.

Particle size also significantly influenced the community structure of fermenting microbiota. Figure 3 shows changes in microbial community structure over 12, 24, and 48 h of *in vitro* fermentation. After 12 h, separations among communities consuming different particle sizes were small; however, by 24 h, the smallest particle size (180–250 μm) began to separate in community structure from the other three bigger sizes, which clustered together. After 24 h, differences began to emerge among the communities fermenting bran particles larger than 250 μm. We observed apparent separation between the 180–250 μm fraction, the 250–300 μm fraction, and the 500–850 μm fraction after 48 h; however, two replicates of the 300–500 μm fraction more resembled the 250–300 μm fraction and one more resembled the 500–850 μm fraction. These data suggested that divergent microbial community structures develop in fermentation of differently-sized maize bran particles.

### Microbial Preferences for Maize Bran Particle Sizes Occurred at Fine Taxonomic Resolution

**FIGURE 4:**
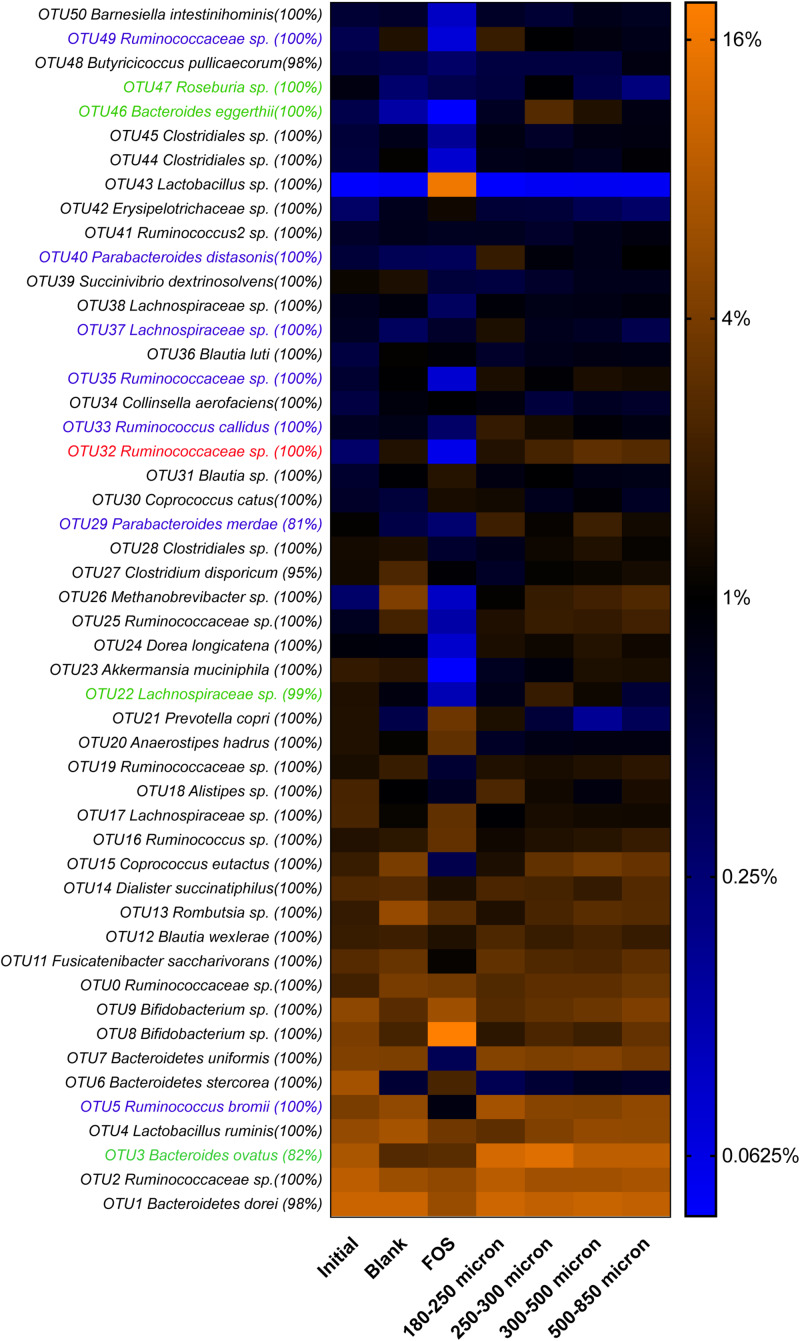
Relative abundances of top 50 OTUs arising in fermentation of maize bran particles diverging in size. OTU abundances were log_2_-transformed for display in the heatmap. Percentage values represent the fraction of reads within an OTU matching the assigned taxonomy of the OTU. Blank represents a no-carbon substrate control, and fructooligosaccharide (FOS) is a fast-fermenting positive control.

**FIGURE 5:**
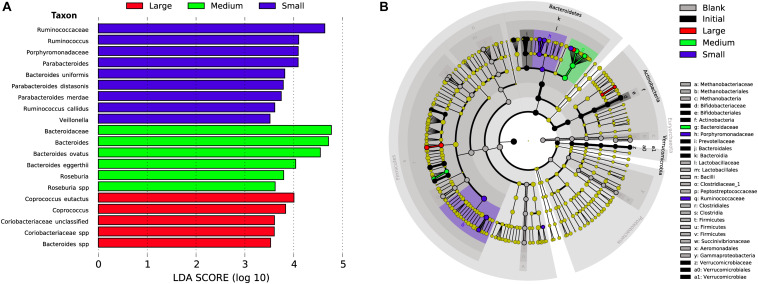
Linear discriminant analysis of taxa representing the small, medium, and large sizes of maize bran. We also included initial and blank communities in the analysis in order to avoid misrepresentation of taxa to size fractions that were overrepresented in controls. **(A)** Taxa with LDA scores > 3.5 in the small (180–250 μm), medium (250–300 μm), and large (500–850 μm) size fractions at 48 after inoculation; linear discriminants of initial and blank conditions are not shown. **(B)** Cladogram showing taxa that are overrepresented in the small, medium, and large maize bran fractions at LDA > 3.5 compared to abundances in the initial inoculum and blank (without carbon substrate).

As in our previous study of fermentation of wheat bran particle size fractions (Tuncil et al., 2018b), in the case of maize bran, we also observed that OTU abundances related to maize bran particle sizes (Figure 4). We used LEfSe to identify statistically-significant linear discriminants of three size fractions: small (180–250 μm), medium (250–300 μm), and large (500–850 μm) (Figure 5). We omitted the 300–500 μm group from the LEfSe, as its replicates were not clearly separated from the smaller and larger size categories. Relative abundances of reads attributed taxonomically corresponded to particle size; small particles generally favored members of families *Ruminococcaceae* and *Porphyromonadaceae* (specifically, genus *Parabacteroides*), and medium particles favored family *Bacteroidaceae*. However, as we observed for wheat brans (Tuncil et al., 2018b), relationships of taxa to maize bran particle sizes were frequently observed at fine taxonomic levels. For example, though members of family *Bacteroidaceae* as a whole were linear discriminants of medium-sized particles (typified by *Bacteroides ovatus*), *B. uniformis* was enriched on small particles and a member of *Bacteriodes* unclassified at the species level was enriched on large particles. Linear discriminants for large particles also included *Coprococcus eutactus* and members of *Coriobacteraceae* unclassified at the genus level.

We also observed species-level preferences for maize bran particle size at the level of individual OTUs. OTUs classified within *Ruminococcaceae* were divided amongst preferences for small and large particles; OTU5 (*Ruminococcus bromii*), OTU33 (*Ruminococcus callidus*), OTU35 and OTU49 (both *Ruminococcaceae* sp.) were linear discriminants for small particles, whereas OTU32 (*Ruminococcaceae* sp.) was enriched on large particles. Similarly, OTUs within family *Lachnospriaceae* showed differential size preferences; OTU37 (*Lachnospiraceae* sp.) preferred smaller particles, whereas OTU22 (*Lachnospiraceae* sp.) and OTU47 (*Roseburia* sp.) were most abundant on medium-size particles. As observed for higher taxonomic classifications, OTUs within *Parabacteroides* also were linear discriminants of small particles and *B. eggerthii* of medium particles. Taken together, these data suggest that preferences for different maize particle sizes are properties of individual species or, potentially, strains of species.

### Maize Bran Particle Size Fractions Differed in Chemical Composition

**FIGURE 6:**
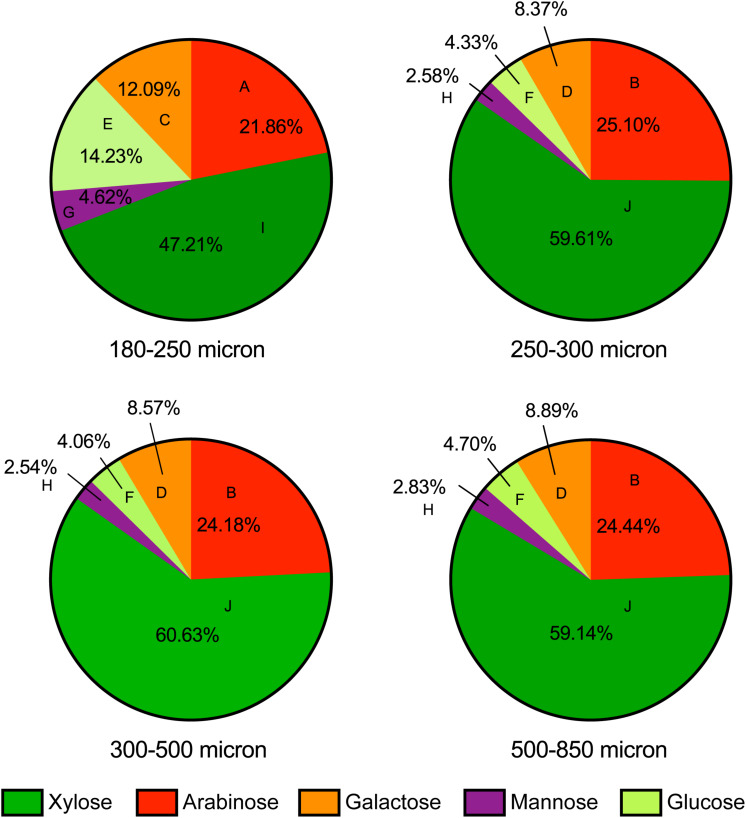
Neutral monosaccharide composition of maize bran particles. Contents of arabinose, xylose, galactose, mannose, and glucose are presented on a mole percent basis. Different letters for each type of sugars indicate the significant differences in the mean value by Tukey’s test and *p* < 0.05.

To attempt to identify mechanisms driving maize bran particle size effects on microbial community structure and function, we examined the chemical compositions of each size fraction to elucidate differences among different maize bran particle sizes. Figure 6 shows the neutral monosaccharide composition of different sizes of maize bran particles. The smallest size (180–250 micron) revealed a significantly higher (*p* < 0.05) proportion of glucose compared to the bigger size fractions. Mannose and galactose proportions were also significantly (*p* < 0.05) higher in the 180–250 micron size range, compared to larger sizes. In contrast, the smallest maize bran particle size exhibited significantly (*p* < 0.05) smaller arabinose and xylose contents, compared to larger sizes. There were no significant differences observed in any of the five sugars (glucose, mannose, galactose, arabinose, and xylose) across the larger three particle sizes of maize bran. Arabinose and xylose are the building blocks of the arabinoxylan polymer, with xylose as the backbone and arabinose forming the branching points (Rumpagaporn et al., 2015), suggesting relatively lower arabinoxylan content in the smallest size fraction. Additionally, the arabinose:xylose ratio was subtly higher in the smallest particle size range. This, combined with statistically-significant elevations in galactose and mannose content, suggest that arabinoxylans within the smallest size fraction may be significantly more branched than in the largest particle sizes.

**FIGURE 7:**
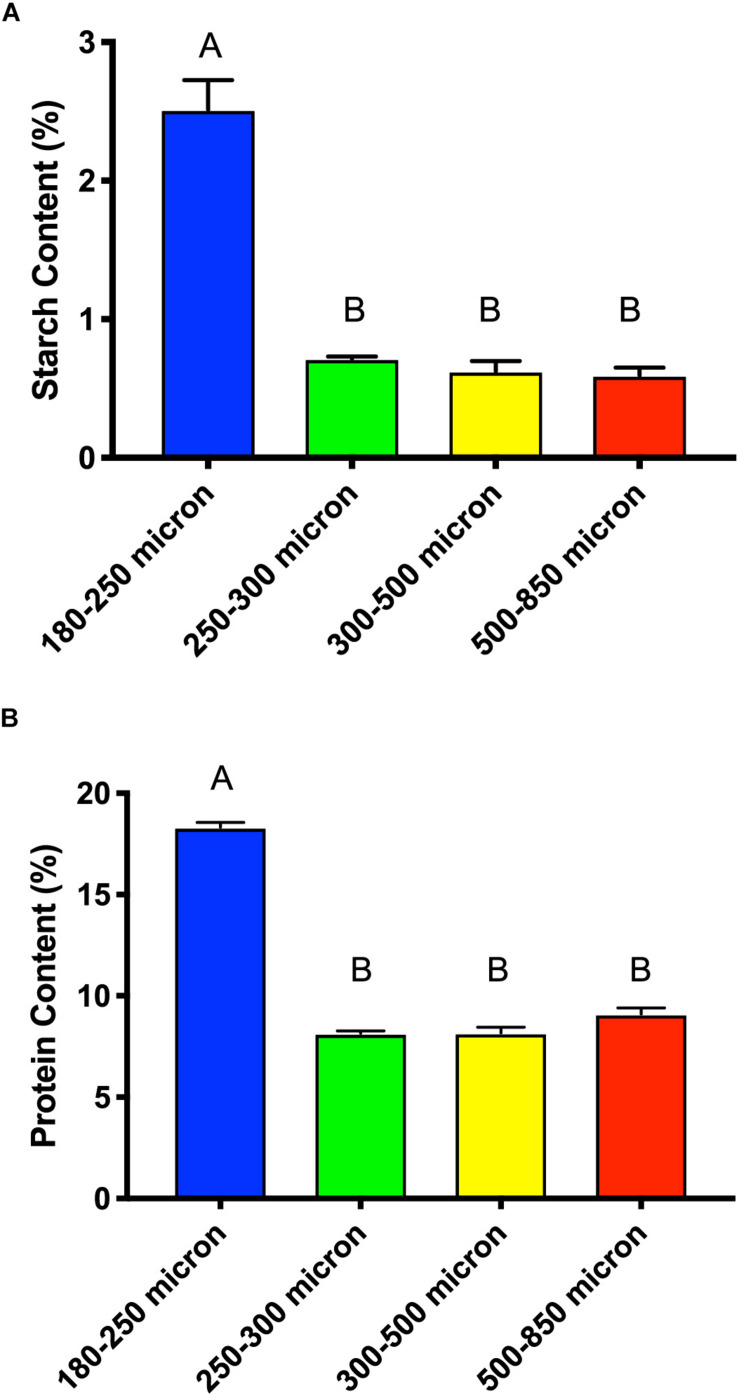
Total starch content **(A)** and protein content **(B)** of different sizes of maize bran after *in vitro* digestion mimicking upper gastrointestinal tract passage. Error bars represent standard error of the mean of triplicate cultures. There is no significant difference between mean values bearing the same letter at *p* < 0.05 using Tukey’s test.

Cellulose and starch are the main sources of glucose in maize brans, thus we tested total starch content (Figure 7A) to determine whether this explained the higher glucose content in the smallest size fraction. As shown in Figure 7A, we measured significantly more starch (about triple) in the smallest particle size, even after *in vitro* upper-GI digestion; differences among the other bran sizes were not significant. This aligns with the neutral monosaccharide composition results in Figure 6, suggesting that the elevated glucose content (with respect to larger particles) is derived from starch and not cellulose. In addition to increases in available starch content, the smallest particle size range contained approximately double the protein of the larger size range (Figure 7B). As with starch, we observed no significant difference among the other three larger maize bran sizes for protein content. It should be noted that the starch assay we employed an enzymatic mechanism, which therefore could be inhibited by physical structuring that separates enzymes from digestible starches. Therefore, physical structure could impact the measured amount of starches from different fractions if protected from enzymatic hydrolysis. In contrast, the protein assay was based upon complete combustion, so measured differences among particle size fractions for protein are insensitive to physical structuring.

## Discussion

The benefits of dietary fiber to the host and metabolite production by microbial fermentation depend both on the fiber type and on the resident microbiota of the host (Rumpagaporn et al., 2015; Tuncil et al., 2017). Along with serving as energy source for the host, SCFAs are known to exert health benefits, including anti-carcinogenic and anti-inflammatory properties (Canani et al., 2011). As many diseases of the colon are localized within the distal colon, reducing gut transit rate and maintaining production of SCFAs throughout colonic transit may suggest this strategy to modulate disease risk. Thus, greater fermentation and SCFA production in the distal colon may reduce the risk of colon cancer and other inflammatory bowel diseases (Lewis and Heaton, 1997). Fermentation of insoluble substrates could provide physical habitats for microbiota and/or potentially support the growth of some slower-growing bacterial populations, increasing microbial diversity. As bran particles are a major source of dietary fiber, manipulation of fermentation properties based upon physical structuring of dietary fiber sources has the potential to influence human health at whole-population scales.

Microbial utilization of dietary fiber is thought to depend on many interrelated structural variables like plant source, sugar type, linkage types, chain length, particle size, anomers, epimers, and association with other compounds (Hamaker and Tuncil, 2014). Amongst these variables, few studies have demonstrated differential gut microbial responses to distinct particle sizes of dietary fibers. A prior study from our laboratory revealed that variously-sized wheat bran fractions resulted in different metabolic outcomes, particularly SCFA production (Tuncil et al., 2018b). 16S rRNA sequencing also revealed that distinct microbial communities arose in fermentation of differently-sized wheat bran fractions (Tuncil et al., 2018b). In this study, we tested our hypothesis that size-dependent fermentation by gut microbiota transcended wheat brans by performing a similar *in vitro* fermentation using identically-sized maize bran particles, so far as they could be recapitulated. As in that study, we found that maize bran size preferences occurred at fine taxonomic resolution, especially for members of genus *Bacteroides*, which differed in their size preferences. Although not broadly selective for members of *Parabacteroides* as observed in this study, we previously observed increases in *Parabacteroides distasonis* in the smallest fractions of wheat brans. These data suggest that, although there were bran-specific variations, similar microbial responses to particle size also occur across brans.

In comparison to wheat bran size fractions, we saw that overall fermentation in terms of production of all three SCFAs (acetate, propionate, and butyrate) was substantially lower for identically sized maize bran fractions (Tuncil et al., 2018b). The relatively low fermentability of maize brans has been well-documented in previous studies by our lab and by others (Mongeau et al., 1991; Tuncil et al., 2018a) and may arise due to the previously-demonstrated low fermentability of the maize arabinoxylan (Rumpagaporn et al., 2015). However, as wheat brans are much more commonly consumed in the Western world, another possible reason for this is that Western gut microbiota have been selected for wheat bran preferences due to exposure rate. Species-level preferences for bran type and particle size intimate that it is possible that size-dependent selective effects for one member of a genus or species, and therefore increases to that species’ relative abundance, might strongly influence overall fermentation behavior in the microbiome.

Furthermore, although the differences in metabolic outcome were more striking for wheat brans, we also observed some size-dependence in SCFA production from different maize bran sizes. This was most obvious for the smallest particle size range, but fermentation of the 250–300 μm range also resulted in elevated production of all three SCFAs. Unlike size-dependent fermentation of wheat brans, however, maize brans tended to ferment to roughly the same SCFA molar ratios, but different total abundances of SCFAs. This also could be explained by the relatively lower fermentability of maize brans compared with other cereals by Western microbiota (Tuncil et al., 2018a). Accordingly, relative abundance changes over time in fermentation of maize brans were substantially smaller than for wheat brans, suggesting that, though organisms displayed differential growth, this growth was smaller in magnitude. Despite the relatively lower metabolism of 300–500 μm maize brans compared with other cereals of identical size ranges, some similarities in microbial responses between our previous study and the present one are obvious. For example, OTUs within *Ruminococcaceae* and *B. ovatus* showed substantial growth on medium-sized bran particles across both studies.

Transit through the upper gastrointestinal tract may significantly impact the chemical composition and microbial response in both bran- and particle size-dependent ways. A previous study in our laboratory observed that the effects of two *in vitro* digestion methods were bran-specific (Tuncil et al., 2018a), suggesting that gastrointestinal passage may differentially impact the fate of brans in the colon. However, it also suggests the possibility that different *in vitro* digestion methods may, in part, influence our microbiome results. In our wheat bran particle size study, we used the Lebet et al. (1998) protocol for mimicking upper gastrointestinal tract digestion, but in this paper employed the Yang et al. (2013) protocol as we determined it was more effective for starch removal from 300 to 500 μm maize bran particles, though less efficient for protein removal (Tuncil et al., 2018a). However, we did not evaluate the efficiency of starch and protein removal across all particle size ranges. Large differences in observed starch and protein content among size fractions suggests (1) differences in anatomical origin of differently sized particles, or (2) that particle size may influence the efficiency of upper GI transit-mimicking *in vitro* digestion methods, which should be rigorously evaluated in future work.

Another key difference between this study and our previous work (Tuncil et al., 2018b) is that, in this case, we milled down the largest size fraction of maize bran into varying sizes to attempt to reduce chemical composition variation among fractions. Regardless, as we observed previously for wheat bran fractions, the smallest particle size (180–250 μm) retained substantially more starch and produced the most SCFAs compared with larger particles. The lack of removal of these starches by *in vitro* digestion suggests that they may be classified type I resistant starches with respect to our procedure. However, it should be noted that the enzymatic method for quantifying starches may be sensitive to starch accessibility, and therefore physical barriers preventing enzyme access to starches in larger particles may influence the result, resulting in undermeasurement of starches present in larger bran particles. However, the glucose content (as determined by acid hydrolysis) is consistent with larger maize particles containing less starch *in toto*. These increased starch contents may preferentially select for members of *Parabacteroides*, which have been shown to be increased in abundance in human feeding trials with resistant starches (Upadhyaya et al., 2016). Yet, it should be noted that particle size effects in this study were not solely dependent upon starch and protein content; medium size particles (250–300 μm) did not substantially differ in their monosaccharide, protein, or starch content from larger fractions, yet the microbial community response was distinct from larger particles. This suggests that there may be other size-dependent resource constraints that influence interaction with gut microbiota.

We also measured protein content and found that the smallest particles also exhibited substantially higher amounts of protein; as this work used a complete combustion method, no accessibility effects would be involved. These data, combined with glucose and starch abundance data, suggest very substantial overall differences in composition of the smallest bran particles compared with larger fractions. This is notable given that the entire size range of particles was generated by milling down the largest fraction (500–850 μm). The three larger fractions, on the other hand, had higher arabinose and xylose contents, which are components of the arabinoxylan polymer (Rumpagaporn et al., 2015) and suggest relatively higher arabinoxylan content in the larger bran particles. Thus, microbial genotypes that possess genes for hydrolyzing these complex, branched arabinoxylan structures might be selected on the larger particle sizes of maize bran on this basis.

Taken together, these data, in concert with our previous work on wheat bran particle sizes, suggest that there are preferential break points within brans that are labile to cyclone milling and that potentially release small sections of endosperm or aleurone adherent to brans as particles that fall within the smallest size range we measured. The high-arabinoxylan portions may be relatively harder to break using these methods. Whether similar preferential break points exist when using other milling methods and whether they partition brans equivalently should be examined in future work to determine whether the choice of milling method influences microbial responses to generated particles standardized across particle size.

Here, we show that different maize bran particle sizes elicit different microbial community structures and metabolic outcomes in *in vitro* fermentation. As similar responses were previously observed for wheat brans (Tuncil et al., 2018b), we therefore suggest that differences in bran physical sizes and structures arising from milling and processing may be a general feature influencing their fermentation by gut microbiota. These data further suggest that particle size is a variable that may be tunable. This shows that the gut microbial communities have varied preference for insoluble fibers based on their sizes. This suggests that not only chemical variations, but also physical variations, like particle size, should be accounted for in the field of fiber-microbiota interaction studies. Furthermore, *in vitro* fermentation experiments, though very important to understand what kinds of interactions between microbiota and food components are possible, are limited in their abilities to predict how these interactions may occur in more-complex environments *in vivo*, where they are subject to host processes. We therefore submit that particle size influences on the gut microbiome should be more thoroughly examined in humans and appropriate animal models to examine their potential microbiome and health effects.

## Data Availability Statement

The datasets generated for this study can be found in the U.S. National Center for Biotechnology Information’s Sequence Read Archive (https://www.ncbi.nlm.nih.gov/sra) as BioSamples SAMN13945813–SAMN13945869 of BioProject PRJNA603892.

## Ethics Statement

The studies involving human participants were reviewed and approved by Purdue University Institutional Review Board Protocol #1701018645. The patients/participants provided their written informed consent to participate in this study.

## Author Contributions

RT, YT, and SL designed the study. RT and YT performed the experiments and analysis of samples. RT and SL performed sequence data and statistical analysis. RT, YT, BH, and SL wrote the manuscript.

## Conflict of Interest

The authors declare that the research was conducted in the absence of any commercial or financial relationships that could be construed as a potential conflict of interest.

## Footnotes

1 https://www.mothur.org/wiki/MiSeq_SOP
